# Estimation of the Gender Ratio of Chickens Based on Computer Vision: Dataset and Exploration

**DOI:** 10.3390/e22070719

**Published:** 2020-06-29

**Authors:** Yuanzhou Yao, Haoyang Yu, Jiong Mu, Jun Li, Haibo Pu

**Affiliations:** 1 College of Information Engineering, Sichuan Agricultural University, Ya’an, Sichuan 625000, China; yaoyuanzhou@stu.sicau.edu.cn (Y.Y.); patton@stu.sicau.edu.cn (H.Y.); jmu@sicau.edu.cn (J.M.); lijun@sicau.edu.cn (J.L.); 2 Sichuan Key Laboratory of Agricultural Information Engineering, Ya’an, Sichuan 625000, China

**Keywords:** aquaculture automation, chicken detection, chicken gender classification, computer vision

## Abstract

The gender ratio of free-range chickens is considered as a major animal welfare problem in commercial broiler farming. Free-range chicken producers need to identify chicken gender to estimate the economic value of their flock. However, it is challenging for farmers to estimate the gender ratio of chickens efficiently and accurately, since the environmental background is complicated and the chicken number is dynamic. Moreover, manual estimation is likely double counts or missed count and thus is inaccurate and time consuming. Hence, automated methods that can lead to results efficiently and accurately replace the identification abilities of a chicken gender expert, working in a farm environment, are beneficial to the industry. The contributions in this paper include: (1) Building the world’s first chicken gender classification database annotated manually, which comprises 800 chicken flock images captured on a farm and 1000 single chicken images separated from the flock images by an object detection network, labelled with gender information. (2) Training a rooster and hen classifier using a deep neural network and cross entropy in information theory to achieve an average accuracy of 96.85%. The evaluation of the algorithm performance indicates that the proposed automated method is practical for the gender classification of chickens on the farm environment and provides a feasible way of thinking for the estimation of the gender ratio.

## 1. Introduction

According to a communiqué [1], China's poultry meat output in 2018 was 19.94 million tons, beef 6.44 million tons, and mutton 4.75 million tons. From a worldwide perspective [2], the consumption of poultry meat also accounts for a large proportion. Therefore, poultry farming, especially chicken farming, is particularly important for food supply. Nowadays, the informationization of the chicken industry receives more and more attention.

**Figure 1:**
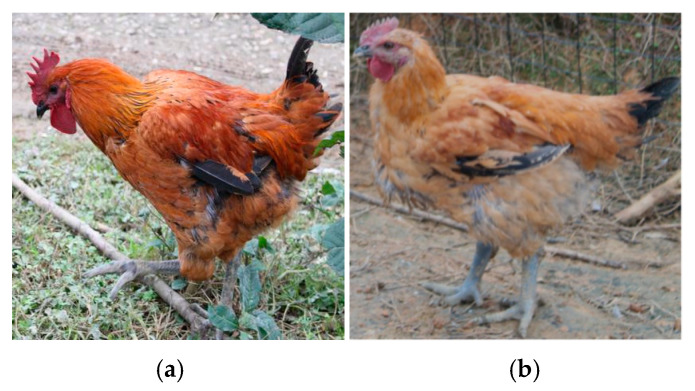
(**a**) shows the appearance of a common rooster, while (**b**) shows the appearance of a hen. The gender difference leads to the difference in appearance. It mainly focuses on three points: (1) the cock's crest is larger while the hen's is smaller; (2) the tail hair of a cock is straight and shaped, while that of a hen is shorter; (3) roosters are more colorful and hens are plainer.

To control the cost of raising chickens and achieve the maximum profit, maintaining a moderate size of flocks is the current best method [3]. When the ratio of roosters and hens in the flock reaches a certain proportion, the reproductive rate and growth health of the flock can reach the optimal state [4]. Moreover, this ratio varies according to the purpose of breeding chickens; for example, the ratio of roosters to hens ranging from 1:8 to 1:9 having the best egg yield and reproduction rate [5,6]. Therefore, it is very crucial for a chicken farm to detect the number of individuals in a flock and count the number of cocks and hens to control the proportion. At present, the main approach for poultry farms to count the number of flocks relies on manual counting, and Figure 1 shows the differences between roosters and hens; however it has the following challenges:

1. Labor intensive—It is time consuming and mentally boring for workers to conduct such operations.
2. Poor performance—It is difficult to count the chickens in motion, and thus can cause repeats or miscalculations.

In recent years, based on sensors and data analytics technologies, many novel chicken flock identification and detection methods have appeared, such as identifying sick chickens in flocks through multispectral images and regions of interest [7]. Dawkins, M.S., et al. [8,9] used an optical flow algorithm to research the flock, including its movement behavior and the entire life cycle measurement. Banerjee, D., et al. [10] used wireless sensors to collect and extract information on flock characteristics, so as to detect flock activity. Wang, L., et al. [11] proposed a network video monitoring system based on embedded technology for a laying hens’ farm. These methods are mainly focused on the detection and identification of chickens’ behaviors and special individuals in the flock, but their algorithms are more qualitative rather than quantitative. Therefore, the accuracy of identification and detection is not very satisfactory.

Deep learning is widely used in industrial scenarios and has been proven to be a very efficient method. Of course, it is also widely used in various agricultural related fields [12]. Some recent studies [13,14,15] prove that deep learning is closely combined with information theory. Deep learning for animal husbandry is also being proposed more frequently, such as by Tian et al. [16], who proposed an automated pig counting method, which used CNN(Convolutional Neural Network) network to obtain the total number of pigs in the whole image by mapping and integrating the density map. In Abu Jwade’s paper [17], deep learning was used to identify and classify four breeds of sheep on the farm, and the experimental results achieved an average accuracy of 95.8 %. Jiang, B., et al. [18] used the improved YOLOv3 network model to detect and identify cow limbs with 99.18% accuracy and 21f/s processing speed. These research works on deep learning show its reliability and efficiency in domesticated animals. According to a recent survey on deep learning in agriculture [10], deep learning has wide application value in poultry breeding.

**Figure 2:**
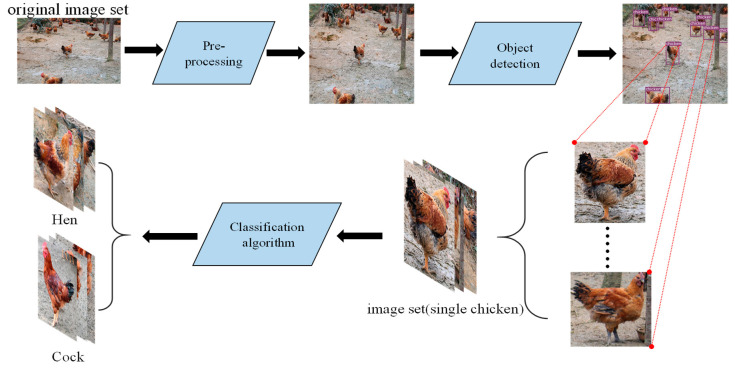
The overall pipeline of the proposed methodology. The original dataset goes through the process of data preprocessing and detection. Then, the single chicken picture goes through the classification network. (In the Supplementary Materials, the dataset of chicken gender classification can be obtained.)

In this paper, we use deep learning to design an automatic detection algorithm, which focuses on the detection of free-range chickens in farms and the classification of chicken gender, and provide an exploration and prospect for the estimation of the gender ratio of chickens on a farm and the direction for intelligent management. By doing so, we can collect the statistical information of gender ratio in the flock and make the reproductive rate and growth health of the flock reach the optimal state, so farmers can achieve the rapid management of farms and more economic profits. Considering that the individuals in chicken flocks are small and dense, and the multi-scale prediction capability of YOLOv3 [19], we employed the YOLOv3 to detect the chicken individuals and then proposed a lightweight classifier for such a small dataset. The overall pipeline of the proposed methodology is shown in Figure 2.

This paper is organized into five sections, including the present one. Section 2 illustrates the principle of the detection and classification network and the related network structure used in the experiment. Section 3 describes the dataset and details the image preprocessing and accuracy evaluation methods. In Section 4, the experimental results and training process of the YOLOv3 object detection model are presented and discussed. The experimental comparison and analysis of different classical classification network models and different processing methods are carried out, respectively. The application and feasibility of the experiment are discussed and prospected. In Section 5, some of the distinctive features of this investigation are highlighted.

## 2. Related Work

### 2.1. YOLOv3-Based Detection Network for Single Chicken

In recent years, great progress has been made in object detection algorithms, which can be divided into two categories. The first is two-stage algorithms, such as R-CNN [20], Fast R-CNN [21] and Faster R-CNN [22], which rely on CNN network to generate Region Proposal first, and then classify and regress on Region Proposal. The other is one-stage algorithms, such as YOLO [23] and SSD [24], which can directly predict the bounding box and class probability from the input image by only using a convolutional neural network structure. However, the early YOLOv1 also has several shortcomings: (1) The input size is fixed, and the output layer is a fully connected layer. So, the YOLO model only supports the same input resolution. Other resolutions need to be scaled to a fixed value; (2) It is not suitable for detecting small objects—although each grid can predict many bounding boxes, only the bounding box with the highest IoU (Intersection over Union) is selected as the object detection output, that is to say, each grid can only predict one object at most. When objects only account for a small the proportion of the picture (e.g., when a herds or birds are shown in images), each grid contains multiple objects, but only one of them can be detected. To address these problems, YOLOv2 [25] was proposed. In YOLOv2, by using a convolution layer, the output layer replaces the fully connected layer of YOLOv1. YOLOv2 cancels all dropout and uses Batch Normalization [26] in the convolution layer. Moreover, YOLOv3 adds multi-scale budget, based on V2 [19]. The YOLOv3 largely improves the detection accuracy, while maintaining a high detection speed [27]. That is why we chose YOLOv3 as our primary model.

### 2.2. Proposed Classifier For Chicken Gender Classification

#### 2.2.1. AlexNet

AlexNet, proposed by Krizhevsky et al. [28], won the 2012 ILSVRC competition with a 16.4% error rate in the Top-5 predictions. It was a major breakthrough in the field of visual recognition and classification tasks, and a historical turning point in the rapidly growing interest in deep learning [29]. AlexNet's network structure is divided into five convolution layers and three full-connection layers. ReLU is successfully used as the activation function of CNN, which illustrates its performance, exceeds Sigmoid in deeper network, and the gradient dispersion problem of Sigmoid in deeper network is successfully solved. AlexNet is the foundation and beginning of deep neural networks, such as VGGNet [30], ResNet [31] and DenseNet [32].

#### 2.2.2. VGGNet

The VGG model [30] is a deep convolutional neural network developed by Computer Vision Combination in Oxford and researchers from Google DeepMind. It explores the relationship between the depth of the convolutional neural network and its performance. By repeatedly stacking 3 × 3 small convolution kernels and 2 × 2 maximum pooling layers, it successfully builds 16 to 19 layers of convolutional neural network. VGGNet won the runner-up in ILSVRC 2014 and was the champion in the positioning project, with an error rate of 7.5% on Top-5. So far, VGGNet is still used to extract image features. Since the model can capture features from images distributed across large and different classes, it has proven to be very useful in dealing with cross-domain image recognition problems [30].

#### 2.2.3. ResNet

ResNet [31] was proposed by Kaiming He, who successfully trained a 152-layer neural network by using the ResNet Unit, which solved the vanishing gradient problem that perplexed previous people. Additionally, he won the championship in ILSVRC 2014. Its error rate on Top-5 is 3.57%, and the parameter is lower than VGGNet. The structure of ResNet can quickly accelerate the training of the neural network, and the accuracy of the model has been greatly improved [31].

#### 2.2.4. Densenet

DenseNet [32] consists of densely connected CNN layers, and the output of each layer is connected with all subsequent layers by dense blocks [32]. Because of this, it forms a tight connection between the layers, and that is how the name DenseNet comes. This concept is effective for feature reuse, it greatly reduces network parameters. DenseNet is separated from the stereotyped thinking pattern, that is, to deepen the network layers [31] and widen the network structure [33] to improve network performance. From the perspective of features, through feature reuse and bypass settings, the problem of gradient vanishing has been alleviated to some extent [32]. Combining information flow and feature reuse assumptions, DenseNet deserves to be the best paper at the Computer Vision Summit of 2017 [34].

All of these network structures are shown in Table 1.

#### 2.2.5. Our classifier

**Figure 3:**
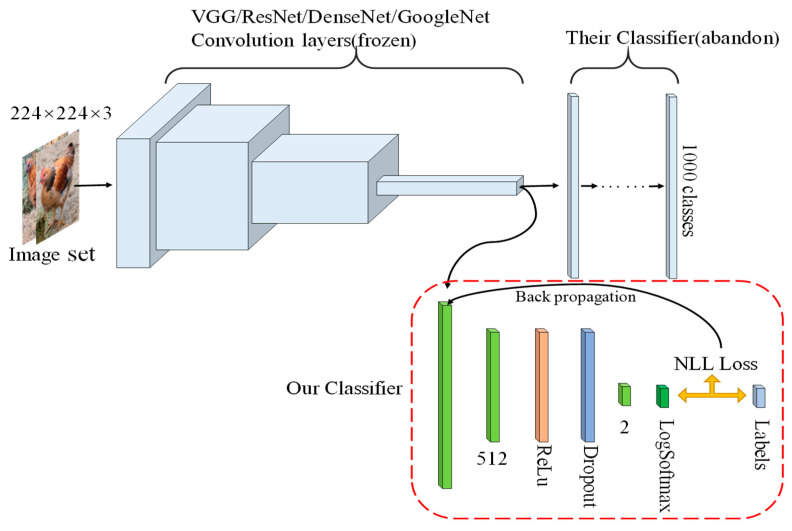
Classification network framework. The classifier is combined with the convolutional layers (the frozen convolutional layers) of other classification networks and discards the original classification network classifier.

The main framework of our classifier is shown in Figure 3. The classifier has only one hidden layer, which contains 512 dimension and is subsequently activated by the ReLu activation function. In order to prevent parameter overfitting and reduce the parameter number in the classifier, we dropout the activated parameters and then the output in two dimensions (classes number). Since this classifier uses NLLLoss [35] as a loss function, a Log Softmax layer needs to be added to the final output. The loss is useful for training a classification problem with N classes. 

## 3. Materials and Methods

### 3.1. Acquisition of Materials

**Figure 4:**
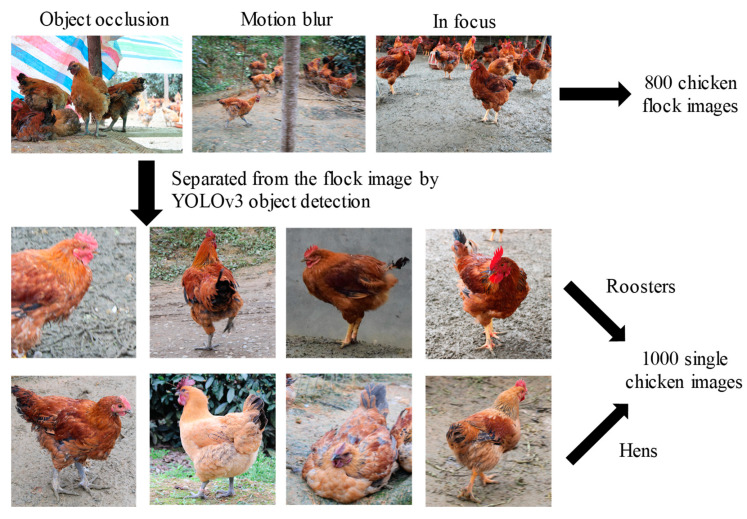
Some sample images from the first chicken gender classification database, which include 800 chicken flock images and 1000 single chicken images. The chicken flock images include three image examples of object occlusion, motion blur and in focus. The classification database of single chicken images was extracted after YOLOv3 object detection.

The images used in this research were taken at Shichang Poultry Co., Ltd., Ya’an City, Sichuan Province. This dataset was taken by a Canon EOS 200D II. The resolutions of the images are 2992 × 2000, respectively. The shooting methods include long-distance shooting and short-distance shooting, and the shooting angle include top-down and parallel. The top-down shooting and long-distance shooting account for most of the dataset, because it is more in line with the angle range of the system's surveillance camera and, thus more practical. Most of the images in the dataset are clear, but some of them are blurred, since the images were captured when the chickens were moving. Therefore, fuzzy image data were added to the dataset to improve model robustness. Additionally, the dataset also contains some of the corresponding behaviors of chickens, including drinking water, eating, waving wings and back information. This not only increases the abundance and diversity of the data, but also makes detection and classification more difficult. Finally, a dataset with a total of 800 images was collected. Pictures that only contain one chicken were further segmented from the detection results. In total, 1000 of them, with obvious gender traits, were selected as the classification dataset, with half cocks and half hens. The detection and classification datasets were both divided by the ratio of 8:2 to build the training set and test set. The collection process of the dataset is shown in Figure 4.

### 3.2. Data Preprocessing

#### 3.2.1. Image Resize and Denoise

Given that the deep learning network framework has fixed input nodes, it was necessary to resize the pictures so that all the image datasets could have a uniform size and also match the size of the network input, because excessively large pictures cause excepts, such as memory overflow. In this paper, we first resized the original picture to 412 × 412 pixels, and entered it into YOLOv3 for detection, then resized the detected single chicken images to 224 × 244, finally inputting them into the classification network for discrimination. The environment in the chicken farm is very complicated. Sometimes it suffers extreme weather, such as rain, so the captured images may be covered by raindrops. In the process of digital image acquisition, encoding, transmission and processing, noise always exists [36]. Due to the advanced equipment we used to collect the dataset, there was less noise in the pictures, while the actual image collection methods in the chicken farm would be through a surveillance camera. Due to the aging of the circuit and the impact of the environment, there was a lot of noise in picture. Therefore, in order to improve the robustness of the algorithm in such circumstances and to fit real scene requirements, a hundred images of the dataset are randomly selected to add salt and pepper noise to simulate the shooting environment. Therefore, filtering the image is an important step to improve the accuracy of detection and classification.

**Figure 5:**
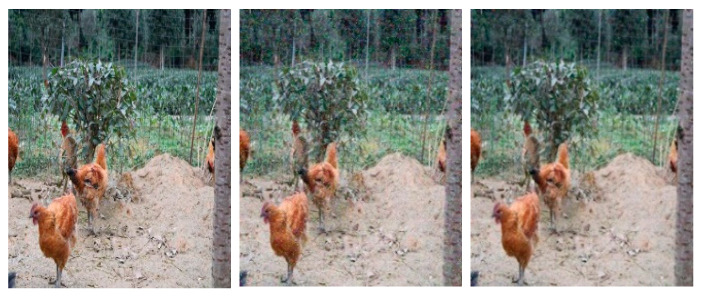
Adding salt and pepper noise and median filtering process.

An effective method for filtering salt and pepper noise is the median filtering, which means replacing the value of one pixel with the middle value in the neighborhood of that pixel. Figure 5 shows some of the filtered image data with salt and pepper noise.

#### 3.2.2. Image Deblurring

**Figure 6:**
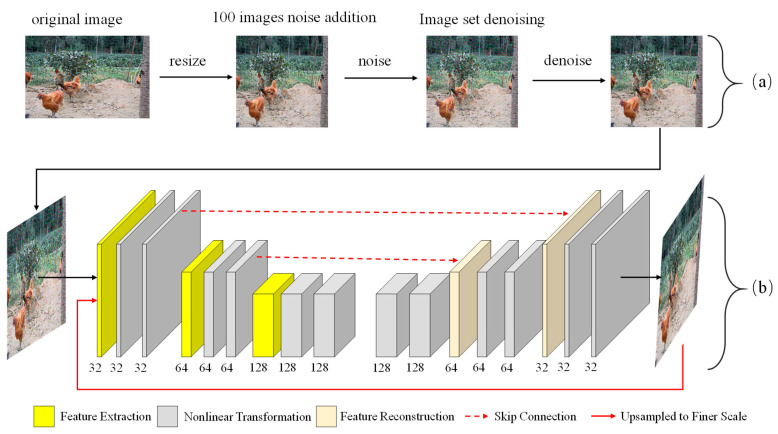
The general framework of the image preprocessing process. After the image is resized, some images are selected to add salt and pepper noise, and then denoising is carried out for the database. The final dataset was inputted into the PSS-NSC defuzzy network to remove motion blur.

Image deblurring is an important issue in computer vision and image processing. Given that an image is likely to be blurred by motion or out of focus (caused by camera shake, fast target movement, or lack of camera focus), the purpose of deblurring is to help it return to an image with clear edge structures and authentic details. In recent years, many related theoretical methods for image deblurring have been proposed. Based on cGAN and "content loss", the DeblurGAN network was proposed, which had the best deblurring effect at that time [37]. In the following improved version, DeblurGAN-v2 builds a deblurring framework for FPN [37], and it can select the backbone network and apply different requirements to select different backbone networks. Tao, X., et al. [38] proposed a scale recurrent network for deblurring tasks. Compared with other learning-based methods, it has a simpler network structure, fewer parameters, and easier training process. There are also many excellent papers on deblurring algorithms [39]. Kupyn, O., et al. [40] proposed a spatiotemporal attention model to integrate information so that adjacent frames in the video can complement with each other. This dataset deblurred on PSS-NSC [40], which proposes a parameter-selective sharing mechanism to build a larger and higher-quality defuzzification network framework. This framework is shown in Figure 6.

**Figure 7:**
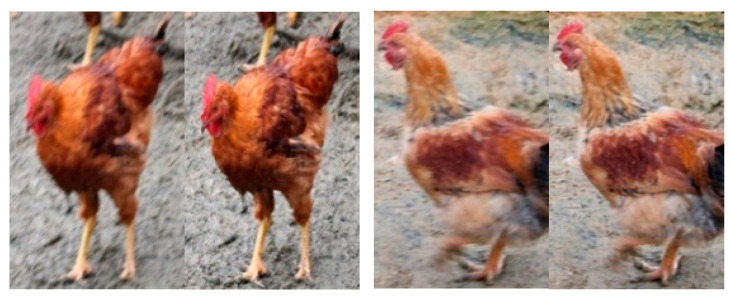
Chicken images before and after the elimination of motion blur.

Its deblurring effect is significant and is also applicable to this dataset, as shown in Figure 7.

### 3.3. Detection and Evaluation Methods

The prediction of the bounding box coordinates in YOLOv3 and the four endpoint formulas of the coordinates are as follows:

(1) $$b_{x}={\sigma (t}_{x})+c_{x}$$

(2) $$b_{y}\;=\sigma (t_{y})\;+\;c_{y}$$

(3) $$b_{w}\;=p_{w}e^{t_{w}}$$

(4) $$b_{h}\;=p_{h}e^{t_{h}}$$

(5) $$Pr(\mathrm{object})\;*\;\mathrm{IoU}(b,\;\mathrm{object})=\sigma (t_{y})$$

$t_{x}$, $t_{y}$, $t_{w}$ and $t_{h}$ in the formula represent the predicted output of the YOLOv3 model. $c_{x\;}$and $c_{y}$ represent the coordinates of each grid cell in the future map. $p_{w}$ and $p_{h}\;$represent the bounding box size before the prediction. $b_{x}$, $b_{y}$, $b_{w}$ and $b_{h}$ are the center coordinates and sizes of the bounding box that we can predict. The loss of coordinates is a squared error loss. In order to judge whether the bounding box predicted by these models has truly detected effective targets, the following judgments will be made:

For the area formed by the four prediction points, we calculated it by IoU. The specific formula is as follows:

(6) $$\mathrm{IoU}=\frac{area(C\cap G)}{area(C\cap G)}$$

In the formula, C represents candidate bound, generated by the target detection model, and G represents the actual annotated ground truth bound. The most common threshold is 0.5—if IoU > 0.5, it is considered to be a correct detection, otherwise it is considered to be a false detection. For those objects that are judged to be correct predictions, they will be used to evaluate the overall accuracy of the final model. For the other evaluation indicators, the definition formulae are:

(7) $$P=\frac{N\left(TruePositives\right)}{N\left(TotalObjects\right)}$$

(8) $$\mathrm{AP}=\frac{\sum Precisions}{N\left(TotalImages\right)}$$

P and AP, respectively, represent the prediction accuracy of each individual image and the overall average accuracy of all the datasets. N represents the number of elements in parentheses.

### 3.4. Experimental Environment

The server platform was configured as an Intel Core CPU I7-9700 K with a 3.60 GHz processor, 16 GB DDR4 2400Mhz memory, 2 TB hard drive capacity, 8 GB NVIDIA GeForce RTX 2080 GPU and the operating system was Windows 10.

## 4. Results and Discussion

### 4.1. Experimental Results

**Figure 8:**
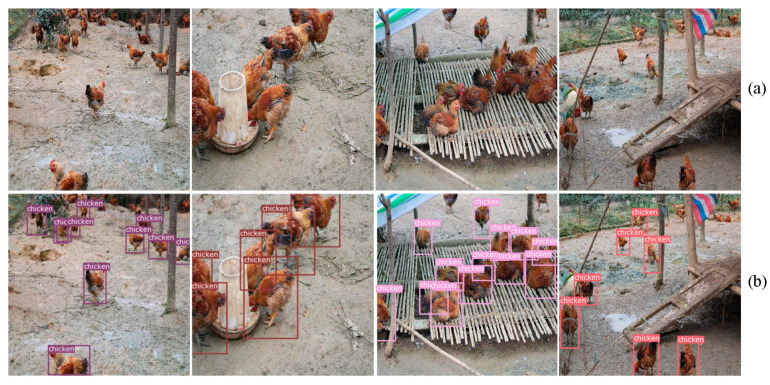
(**a**) The original image. (**b**) Detection results of the chicken flock images. According to the detection visualization effect, it can be shown that YOLOv3 performs well in the detection of a single chicken in the flock. Especially for the flock with severe occlusion, it can still accurately conduct the detection of a single chicken.

**Figure 9:**
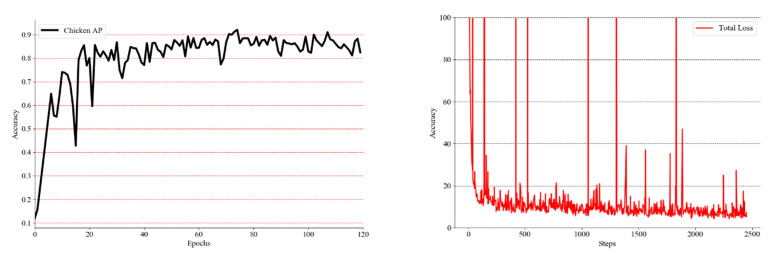
The figure on the left reflects the change in AP value of chicken with the training process, while the figure on the right shows the change in total loss of YOLOv3 with the training process.

Some results of the detection network YOLOv3 are shown in Figure 8, and Figure 9 exhibits the experimental results and the training process on the framework in this dataset. The highest detection accuracy of YOLOv3 was 92.23%. For the gender classification, 1000 pictures with the main features of the chicken were selected as classification datasets. Different network frameworks were selected for experiments to find the most suitable classification convolution framework for the designed classifier. For the classification of such a small dataset, we loaded pre-trained models for transfer learning.

Additionally, the following are the main experimental contributions:

(1) Experiments on the application of YOLOv3 detection algorithm in a dataset that contains 800 chicken flocks.
(2) Based on the test results, 1000 single chicken pictures with obvious features were selected to form a classification dataset.
(3) A lightweight classifier, suitable for small dataset, was designed. Traditional classification networks perform well for a large dataset ImageNet, but it is difficult to handle the classification of small datasets.
(4) The original classifiers of VGG-16, VGG-19, ResNet-18, ResNet-34, DenseNet-121 and AlexNet were discarded, and the feature map after convolution was inputted into the designed classifier for classification experiments.
(5) The above network convolution layers were frozen, and only the classifier was optimized and compared with the optimization of the overall network
(6) It was observed whether BN (Batch Normalization) improves network generalization and accuracy for this dataset after adding BN to VGG-16 and VGG-19 in the experiment.

**Figure 10:**
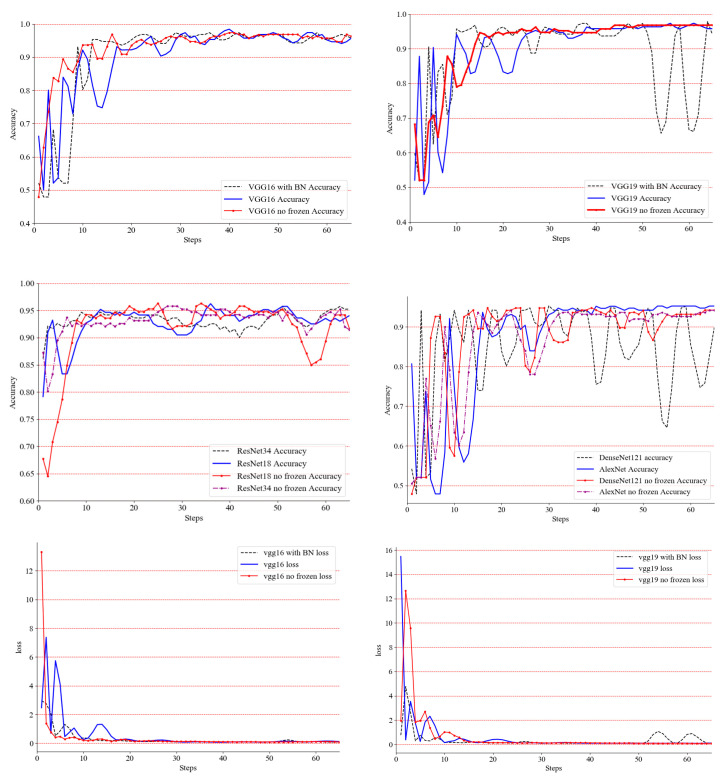

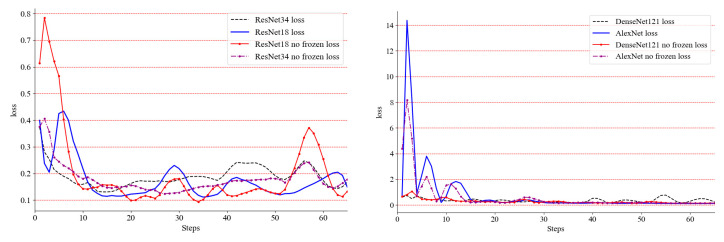
The classification algorithm training progress through 5 epochs (65 steps).

The classification algorithm and detection algorithm were developed based on the deep learning language framework, Torch. YOLOv3’s training parameters and classification training parameters are shown in Table 2. The classification experiment results of a single chicken image set are shown in Table 3, and the training process is shown in Figure 10.

According to the observation in Figure 10, we can draw the following conclusions:

- After the above experiments, for classification networks, such as VGG-16, VGG-19, ResNet-18, ResNet-34, DenseNet-121 and AlexNet, the accuracy and loss of the early training process are severely oscillated, but the late convergence effect is better.
- VGG-16, with or without BN, under the optimized classifier only or global training, gave better results and will converge to a higher classification accuracy. Its structure is suitable for the designed classifier and dataset.
- DenseNet-121 did not perform well in global optimization and it still fluctuated severely in experiments only under the optimized classifier in the later period. After preliminary analysis, its deep network framework is not suitable for such small datasets. The AlextNet network converges well eventually, and its performance in the classifier is also ideal.
- VGG-19 is not suitable for adding BN on this dataset. After adding BN, it oscillates severely. Experiments under global optimization and experiments that were only under the optimized classifier performed well in accuracy and convergence. It proves that VGG-19’s global optimization is the best classification network, whose accuracy converges to 96.85%.
- ResNet-18 and ResNet-34 have higher precision in the later stage, but the final convergence effect is not ideal, and oscillation still exists.

**Figure 11:**
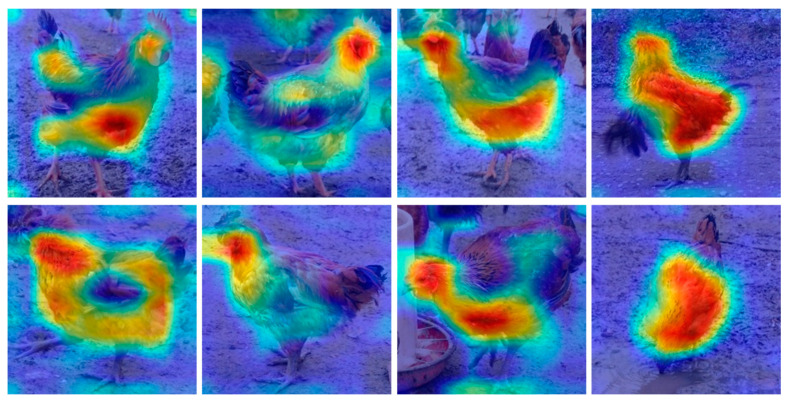
Grad-CAM of VGG-19.

The Grad-CAMs [41] of the developed models were generated, as shown in Figure 11. VGG-19 was chosen as the model to be visualized because VGG-19 reached the highest accuracy and the most convergence. The Grad-CAMs displayed that the model focused on the same parts of the chicken bodies in most of the cases. Whether cock or hen, VGG-19 focuses on the head. Therefore, the model is mainly classified by the head and trunk regions of the chicken.

In the actual application of a chicken farm, there are several application requirements, as follows:

(1) The density of free-range chickens is low, so there is no chicken overlap problem.
(2) In broiler farms, there are generally only two chicken species involved, namely Sanhuang chicken and Taoyuan chicken. This dataset is taken from these two species. Most breeds of chickens only change color according to breed differences, but gender differences still exist and are the same. Therefore, the problem that the classifier is not universal, due to the large in-class distance, is not involved.

### 4.2. Discussion and Outlook

This paper explores the gender ratio of chickens in farms and explores a method to estimate the gender ratios of chickens automatically. We can detect individual chickens in the corresponding images, and then make gender judgments through the classifier we designed, so as to obtain the gender ratios of chicken flocks. As for the feasibility of this approach, we discuss the following:

(1) In terms of processing accuracy, the source of object detection is the image collected by a single camera on the breeding environment. Due to the fixed position of the camera and the single collection angle, the information of the overall breeding environment cannot be obtained by a single camera, so the information acquired is single and limited. The movement of chickens may also lead to a huge error between the information obtained by a single camera and the actual situation. Therefore, farms should consider deploying multiple cameras in the breeding environment to conduct all-round and multi-angle information collection in the breeding environment, and to calculate the overall situation of the chicken farm by integrating the information taken by multiple cameras. Considering that the image information collected by the camera may be overlapped by a large number of chicken individuals, although it may interfere with the object detection (which does not mean that the accuracy will decrease directly), the purpose of our study was to make a judgment on the gender ratio in the chicken population, rather than by simply counting. In general, although there may be some individuals missing from the target test, the small number of these individuals and their gender ratio- are approximately the same, as the population as a whole will not have a significant impact on the gender ratio of the overall flock.
(2) In terms of processing speed, in order to meet a farmer’s need to know at any time, real-time processing of the images collected by the camera is required. This is also the reason why we initially considered using an object detection model instead of instance segmentation or semantic segmentation and other models (the latter two are relatively slow in processing speed). Among the many object detection models, we considered using YOLOv3 model for processing, which is also an advanced model in the one-stage methods in object detection fields. Compared with the two-stage method, the former has a higher processing speed under the same hardware conditions. Compared with other one-stage methods (such as YOLOv2), the related reasons have been described in Section 2.1, which will not be repeated here.

Based on the above discussion, we believe that our proposed method is an effective exploration and discovery of the development of the management of automatic breeding systems.

## 5. Conclusions

In this study, deep learning technology was applied to the detection and classification of individual chickens, and proposed a high-precision algorithm for chicken detection and gender classification. Firstly, a dataset containing 800 chicken flock images was collected. Then, 1000 single chicken pictures with obvious traits were extracted by conducting the YOLOv3 detection algorithm on the original collected image to form a classified dataset. To our best knowledge, this is the world’s first dataset about chicken gender. For this small dataset, we designed a lightweight classifier, and combined it with convolutional layers of VGG-16, VGG-19, ResNet-18, ResNet-34, DenseNet-121 and AlexNet for experiments. Additional, transfer learning and frozen convolutional layers were used to test the classification performance of the classifier. Experimental results indicate that the classifier performs well and, specifically, VGG-19 has the best performance, whose classification accuracy is up to 96.85%. Meanwhile the detection accuracy of YOLOv3 is up to 92.23%. Therefore, the accuracy of single chicken detection and gender classification meet the requirements of practical applications. Aiming at the experimental results, we discussed the realization method of the automatic estimation of the gender ratio of chickens in the farm, which provided the train of thought for the automatic management of chicken farms. In the near future, we tried to explore different detection algorithms to determine the optimal one for the uncertain scenarios of the flock.

## Figures and Tables

**Table 1:** The classification network structure mentioned in this paper. In this paper, a total of four classical classification networks were used. The VGG and ResNet classification networks selected 16 layers, 19 layers, 18 layers and 34 layers, respectively, and the detailed structure of each network is presented in the table.

	AlexNet	VGG		ResNet		DenseNet
	8-layer	16-layer	19-layer	18-layer	34-layer	121-layer
Conv1_x	11×11,96	3×3,64 3×3,64	3×3,64 3×3,64	7×7,64 stride 2		7×7,64 stride 2
	3×3 overlapping pool,stride 2	3×3 max pool, stride 2				3×3 max pool,stride 2
Conv2_x	5×5,256	3×3,128 3×3,128	3×3,128 3×3,128	$\left(\begin{array}{c} 3\times 3,64\\ 3\times 3,64 \end{array}\right)\times 2$	$\left(\begin{array}{c} 3\times 3,64\\ 3\times 3,64 \end{array}\right)\times 3$	$\left(\begin{array}{c} 1\times 1,32\\ 3\times 3,32 \end{array}\right)\times 6$ 1×1,32
	3×3 max pool,stride 2	3×3 max pool, stride 2				2×2 average pool,stride 2
Conv3_x	3×3,384	3×3,256 3×3,256 3×3,256	3×3,256 3×3,256 3×3,256 3×3,256	$\left(\begin{array}{c} 3\times 3,128\\ 3\times 3,128 \end{array}\right)\times 2$	$\left(\begin{array}{c} 3\times 3,128\\ 3\times 3,128 \end{array}\right)\times 4$	$\left(\begin{array}{c} 1\times 1,32\\ 3\times 3,32 \end{array}\right)\times 12$ 1×1,32
		3×3 max pool, stride 2				2×2 average pool,stride 2
Conv4_x	3×3,384	3×3,512 3×3,512 3×3,512	3×3,512 3×3,512 3×3,512 3×3,512	$\left(\begin{array}{c} 3\times 3,256\\ 3\times 3,256 \end{array}\right)\times 2$	$\left(\begin{array}{c} 3\times 3,256\\ 3\times 3,256 \end{array}\right)\times 6$	$\left(\begin{array}{c} 1\times 1,32\\ 3\times 3,32 \end{array}\right)\times 24$ 1×1,32
		3×3 max pool, stride 2				2×2 average pool,stride 2
Conv5_x	3×3,256	3×3,512 3×3,512 3×3,512	3×3,512 3×3,512 3×3,512 3×3,512	$\left(\begin{array}{c} 3\times 3,512\\ 3\times 3,512 \end{array}\right)\times 2$	$\left(\begin{array}{c} 3\times 3,512\\ 3\times 3,512 \end{array}\right)\times 3$	$\left(\begin{array}{c} 1\times 1,32\\ 3\times 3,32 \end{array}\right)\times 16$
	3×3 max pool,stride 2	3×3 max pool, stride 2		average pool		7×7 global average pool, stride 2
Classification layer	fc 4906	fc 4906		fc 1000		fc 1000
	fc 4906	fc 4906				
	fc 1000	fc 1000				
	softmax	softmax		softmax		softmax

**Table 2:** Training hyper parameters.

	Learning Rate	Batch Size	Maximum Number of Epochs	Iterations Per epoch	Momentum	Decay
YOLOv3	0.01	4	120	160	0.9	0.0005
Classifier	0.01	64	5	13	-	-

**Table 3:** The classification accuracy of each epoch was based on 14 experimental methods.

	Process Mode	Epoch1 (%)	Epoch2 (%)	Epoch3 (%)	Epoch4 (%)	Epoch5 (%)
AlexNet	frozen	55.88	89.33	94.17	94.71	95.24
	not frozen	63.41	86.67	93.70	91.98	92.61
VGG-16	frozen	81.68	96.23	95.31	96.82	95.16
	with BN	**95.29**	**96.28**	**97.34**	**96.82**	95.16
	not frozen	93.75	93.75	95.76	96.82	95.73
VGG-19	frozen	88.54	92.53	93.60	96.33	**96.85**
	with BN	95.26	95.78	96.80	95.71	85.43
	not frozen	83.34	95.73	94.66	96.25	96.80
ResNet-18	frozen	94.17	92.61	96.25	95.16	93.55
	not frozen	93.62	95.29	95.21	94.77	89.33
ResNet-34	frozen	94.12	94.17	92.55	93.67	94.64
	not frozen	92.68	94.19	94.19	93.67	94.25
DenseNet-121	frozen	81.60	94.12	94.71	83.89	85.53
	not frozen	92.55	94.74	93.70	93.70	93.18

## Supplementary Materials

The following are available online at https://drive.google.com/drive/folders/1eGq8dWGL0I3rW2B9eJ_casH0_D3x7R73, The dataset of chicken gender classification used in the experiment can be obtained here. As we are still doing more research on the images of chickens in the farm, we will upload our chicken flock dataset to the same sharing link later.
